# Reflection: AI as a catalyst for change in postgraduate medical education

**DOI:** 10.4102/jcmsa.v4i1.413

**Published:** 2026-06-25

**Authors:** Sumaiya Adam, Vanessa C. Burch

**Affiliations:** 1 Department of Obstetrics and Gynaecology, School of Medicine, Faculty of Health Sciences, University of Pretoria, Pretoria, South Africa; 2 Colleges of Medicine of South Africa, Cape Town, South Africa

**Keywords:** artificial intelligence, medical education, bedside teaching, student-centred, critical thinking, never-skilling

## Abstract

Artificial intelligence (AI) presents a timely opportunity to modernise postgraduate medical education, which has remained largely unchanged despite rapid technological advancements. AI can streamline administrative tasks, personalise learning and support critical thinking, allowing educators to refocus on mentorship and bedside teaching. However, if used uncritically or introduced too early as a substitute for clinical reasoning, AI may contribute to the risk of ‘never-skilling’, where students fail to develop the foundational clinical skills and independent judgement needed for safe practice. Integrating AI and other digital tools into clinical environments fosters authentic, technology-enabled learning while promoting digital professionalism. A collaborative, environmentally conscious approach across disciplines and institutions is essential to ensure equitable, sustainable adoption. The thoughtful use of AI can transform postgraduate medical education into a more efficient, student-centred system, aligned with the realities of modern healthcare.

## Introduction

In just a few generations, the world has transformed. Cars now drive themselves, televisions stream content on demand and mobile phones have evolved into powerful handheld computers. Yet, despite these advances, the physical and conceptual spaces of postgraduate medical education remain strikingly similar to those of a century ago.^1^ Our classrooms, ward rounds and even assessment methods still reflect a static model of learning, even as the world around us continues to change dramatically. Artificial intelligence (AI) is an invitation to change that. It is not a passing trend; it is already part of our daily lives and will increasingly shape the practice of medicine. Our students are using it, and so are we. The question is not whether AI belongs in postgraduate medical education, but how we will embrace it thoughtfully and purposefully.^2^

For us, the way forward is to intentionally incorporate AI into postgraduate medical education, with care and purpose. Artificial intelligence should support educators, not replace them. It should bring learning closer to authentic clinical practice, to be used with guidance and critical appraisal and be introduced in ways that are equitable and realistic across various training settings.

## Working smarter, not harder

For us, one of the greatest promises of AI is that it can help us work smarter. It can automate repetitive tasks, generate teaching materials, summarise literature and even support feedback and assessment.^2,3,4^ Instead of fearing that AI might replace us, we should see it as a tool that frees us to focus on what truly matters: mentoring, clinical reasoning, and the human connection that sits at the heart of medicine.

Artificial intelligence can make learning more student centred by allowing each student to explore material at their own pace, ask questions in new ways and receive tailored support. It encourages curiosity, supports critical thinking and allows students to interrogate knowledge more deeply, thus preparing them for a future in which clinical decision-making will always involve a partnership between human judgement and digital intelligence.^5^

However, this optimistic outlook must be balanced by acknowledging the associated risks. Artificial intelligence systems can generate convincing but inaccurate outputs.^6,7,8^ Algorithmic bias, especially when combined with uncritical use, may reproduce historical inequalities and discrimination rather than challenge them.^9,10^

This is why AI must be used to strengthen critical thinking, not bypass it. Our teaching methods also need to adapt. We can no longer teach learning strategies as if students are studying in a pre-AI world. Instead, we need to help students use AI in ways that support active recall, questioning, reflection and clinical reasoning, rather than passive consumption or over-reliance on automated answers.^11^

Data privacy, patient confidentiality, transparency of AI outputs and accountability for decisions must therefore be treated as core educational concerns rather than technical afterthoughts. Students should be taught not only how to use AI tools but also how to question them, verify their outputs, recognise their limitations and preserve human judgement in clinical decision-making.^12^

## Reclaiming the bedside

Paradoxically, we believe that using AI well can help bring us back to the bedside, and this is no longer optional. If students learn medicine mainly through screens, summaries, dashboards and automated outputs, there is a real risk that they may never fully develop the clinical skills and independent reasoning that are built through repeated patient contact.^13^ This concern has recently been described as ‘never-skilling’: the failure to develop foundational competencies during formative training when AI substitutes for the cognitive effort required to build independent clinical reasoning.^14^ The consequences are serious: loss of confidence in clinical examination, weaker clinical reasoning, reduced ability to recognise subtle changes in a patient and over-reliance on technology to make sense of what is in front of them.

If AI can reduce the time we spend on administrative work, question writing, marking and data analysis, then that time should be deliberately reclaimed for bedside teaching, where medicine truly comes alive. This does not mean keeping technology away from patients. Rather, it means bringing AI and other digital tools into bedside teaching in a deliberate and visible way, so that students learn how to use them in real clinical contexts. This includes interpreting data, questioning outputs, recognising when a recommendation does not fit the patient and testing their own reasoning against what the technology suggests. Digital tools are already part of everyday clinical practice, from point-of-care ultrasound devices and handheld Dopplers linked to mobile apps, to electronic medical records, digital early-warning score systems and clinical decision-support platforms.^15,16^ When these tools are intentionally incorporated into bedside teaching, students learn not only how to use them but also when to trust them, when to question them and why clinical judgement must remain central.

Students are already using these tools, whether formally endorsed or not. Our responsibility is to help them become more discerning in which tools they choose, how they use them and how they interpret their outputs. Such activities move AI from a hidden background tool into the visible space of clinical reasoning and reflective practice.^3,5^ They also support a shift from a purely ‘just-in-case’ model of learning, where knowledge is accumulated for possible future use, towards a more ‘just-in-time’ model, in which knowledge is accessed, tested and applied in response to the patient’s immediate clinical context.^17,18^

At the bedside, the patient must remain at the centre of the learning encounter. Hennus et al. argue that bedside teaching should be understood as a triad between teacher, learner and patient, with the patient playing an active rather than passive role. This is especially important in an AI-enabled clinical environment, where there is a risk that students may focus more on outputs than on the person in front of them.^19^ Bedside teaching gives students the opportunity to listen to the patient’s story, recognise emotional cues, clarify priorities and see how these human details shape clinical reasoning and shared decision-making. Artificial intelligence may help us organise information, curate knowledge or support reasoning, but it cannot teach presence, empathy, trust or professional judgement on its own. For that, we still need the bedside.

Artificial intelligence can also support the teaching process itself. Parmar et al. describe an AI-supported ‘Rounds Copilot’ system that captures spoken bedside teaching and converts it into structured learning materials. In their example, a simulated 15-min pericarditis teaching encounter was converted within 3 min into seven teaching points, evidence-versus-clinical-pearl classifications and five formative multiple-choice questions.^20^ This offers a practical example of how AI could preserve spontaneous bedside teaching, reduce the pressure on students to take notes during patient care and help them distinguish guideline-backed recommendations from experiential clinical wisdom.

These examples matter because bedside teaching occurs where students develop skills that cannot be acquired from screens alone. Ravichandran and Saravanan emphasise that bedside teaching allows students to observe clinical reasoning, communication, compassion, empathy, subtle signs, tone of voice, touch during examination, teamwork and ethical challenges in real clinical contexts.^21^ In this way, digital tools should not replace bedside teaching but help us make it more deliberate, reflective and durable. Used well, they can support students to observe more carefully, question more deeply, distinguish evidence from opinion and remain anchored to the patient in front of them.

Teaching in this way mirrors the actual workflow of modern clinicians, who increasingly rely on a combination of clinical skills, digital data and algorithm-supported insights to make informed decisions. Whether it is tracking patient trends on digital dashboards, viewing imaging on handheld devices, using real-time risk calculators or showing how AI-generated summaries can complement clinical judgement, these activities allow students to experience technology as a natural and cohesive part of the clinical environment.^16^ Rather than simply teaching students to use digital systems, educators can teach them to interrogate these systems, integrate them with bedside findings, recognise when outputs may be incomplete or misleading and remain accountable to the patient in front of them.

By normalising the presence of digital tools at the bedside, educators can also model critical digital professionalism: how to maintain patient trust, protect confidentiality, apply ethical judgement and avoid over-reliance on automated outputs. This type of teaching fosters digital literacy, situational awareness and clinical humility. It also reinforces a simple but important message: technology can enhance human expertise, but it does not replace it.^1,5^ Most importantly, it ensures that learning is grounded in authentic clinical contexts. Students develop competence not only in traditional bedside examination, but also in the blended, technology-enabled care that defines contemporary healthcare systems. This prepares them to deliver care that is safe, evidence-based, efficient and aligned with the digital transformations shaping the future of medicine.

## Building smarter systems together

We must also be thoughtful about how we use AI. It carries environmental costs, and access remains uneven, which means our approach must be both responsible and deliberate.^22,23^ This moment calls for us to break down traditional silos, not only between institutions but also between disciplines, professions and sectors. By cultivating communities of practice that bring together clinicians, educators, technologists, data scientists and students, we can co-design AI tools and digital solutions that genuinely meet educational and clinical needs. Sharing tools, datasets and best practices across training institutions is essential to avoid duplication, reduce resource burden and ensure that innovation benefits everyone rather than a select few.

Interdisciplinary education should also be encouraged, so that students can appreciate how technological advances intersect with ethics, public health, communication and systems thinking.^24^ This collaborative approach also encourages a climate-conscious mindset, reminding us that the rapid growth of digital technologies must be balanced with careful stewardship of energy use and environmental impact.^22^

In low-resource settings, AI adoption should not be framed as a race towards the newest or most sophisticated tools, but as a phased, context-sensitive process aimed at reducing rather than perpetuating inequity. Institutions may begin with low-cost, high-value applications such as faculty development, AI literacy, assessment support, feedback generation and structured literature summaries before progressing to higher-risk uses such as clinical decision support. This staged approach is important because delayed, uneven or poorly governed AI adoption could create a new digital divide, leaving under-resourced training platforms further behind well-resourced institutions. To avoid this outcome, AI integration should be supported by shared institutional policies, technical guidance, educational resources and mechanisms for evaluating whether tools are fair, appropriate, valid, effective and safe in local contexts. Open educational resources, regional collaboration and careful selection of affordable, secure and locally relevant technologies will be essential to ensure that AI strengthens postgraduate education as a collective capacity-building tool rather than fragmenting it along existing lines of privilege.^25^

Although evidence from low- and middle-income countries remains limited, emerging examples suggest that AI is already beginning to shape health professions education and continuing professional development in these settings. Studies from Uganda have shown that medical students and faculty are already using ChatGPT and other AI tools, highlighting both the opportunities for learning support and the urgent need for AI literacy and responsible-use guidance.^26,27,28^ In southern Africa, discussions on the use of AI and extended reality in health professions education have similarly emphasised the importance of context-sensitive implementation, ethical awareness, confidentiality and educator preparedness.^29^ In Senegal, AI-supported continuing education initiatives are being explored to improve access to clinical guidelines and standard operating procedures for health practitioners.^30^ These examples do not yet provide definitive evidence of improved postgraduate training outcomes, but they illustrate why phased implementation, governance and local evaluation are essential in resource-constrained settings.

Together, collaboration, shared resources, interdisciplinary learning and environmental responsibility can form the foundation for a more equitable and sustainable integration of AI into postgraduate medical education. Responsible integration will also require institutional governance frameworks that define acceptable use, protect patient data, clarify accountability and guide disclosure of AI use in teaching, assessment and scholarly work. Artificial intelligence literacy should become part of faculty and student development, including basic understanding of prompt design, data privacy, bias, verification of outputs and the ethical limits of automation. Oversight structures should include clinicians, educators, students, information governance experts and institutional leaders so that AI use remains educationally sound, clinically safe and socially accountable.^31,32^

## Conclusion

We are at a rare moment of possibility. For too long, many of our teaching environments have remained static while the world around us has changed. Artificial intelligence offers us the chance to do more than digitise old methods; it allows us to reimagine postgraduate education as more adaptive, clinically authentic and student centred.

Yet the task is not simply to adopt AI, but to use it wisely. Its value will depend on whether we use it as part of a deliberate educational strategy: to augment educators, strengthen bedside learning, deepen independent clinical reasoning, promote digital professionalism, protect patients and reduce rather than widen inequities. The future of postgraduate medical education should therefore be shaped by thoughtful integration, interdisciplinary governance, environmental responsibility and a continued commitment to the human relationships at the heart of medicine.

Artificial intelligence is here. Our students are already using it. Our responsibility now is to teach them to use it with curiosity, caution, compassion and critical thinking.

## References

[1] Tokuç B, Varol G. Medical education in the era of advancing technology. Balkan Med J. 2023;40(6):395–399. 10.4274/balkanmedj.galenos.2023.2023-7-79 37706676 PMC10613744

[2] Mir MM, Mir GM, Raina NT, et al. Application of artificial intelligence in medical education: Current scenario and future perspectives. J Adv Med Educ Prof. 2023;11(3):133–140. 10.30476/JAMP.2023.98655.1803 37469385 PMC10352669

[3] Zarei M, Mamaghani HE, Abbasi A, Hosseini MS. Application of artificial intelligence in medical education: A review of benefits, challenges, and solutions. Med Clín Práct. 2024;7(2):100422. 10.1016/j.mcpsp.2023.100422

[4] Reyna J. The potential of Google NotebookLM for teaching and learning. Presented at: eLearn 2025; 2025 Oct 13–16 [cited 2026 May 15]; Bangkok, Thailand. Available from: https://www.academia.edu/143048105/2025_The_Potential_of_Google_NotebookLM_for_Teaching_and_Learning.

[5] Sikri D, Nuguri V. From notes to networks – A three-dimensional framework with integrated curation for using Google NotebookLM in health professions education. Med Teach. 2025;18:1–5. 10.1080/0142159X.2025.2533408 40679244

[6] Park PS, Goldstein S, O’Gara A, Chen M, Hendrycks D. AI deception: A survey of examples, risks, and potential solutions. Patterns (NY). 2024;5(5):100988. 10.1016/j.patter.2024.100988 PMC11117051 38800366

[7] Cross JL, Choma MA, Onofrey JA. Bias in medical AI: Implications for clinical decision-making. PLoS Digit Health. 2024;3(11):e0000651. 10.1371/journal.pdig.0000651 39509461 PMC11542778

[8] Teng D, Tan L, Cao Q, et al. Impact of AI misinformation on diagnostic accuracy and confidence calibration in novice medical students. NPJ Digit Med. 2026;9:356. 10.1038/s41746-026-02547-z 41844809 PMC13144481

[9] Norori N, Hu Q, Aellen FM, Faraci FD, Tzovara A. Addressing bias in big data and AI for health care: A call for open science. Patterns (NY). 2021;2(10):100347. 10.1016/j.patter.2021.100347 PMC8515002 34693373

[10] Kharitonova YS, Savina VS, Pagnini F. Artificial intelligence’s algorithmic bias: Ethical and legal issues. Bulletin of Perm University Herald. Juridical Sciences. 2021;(53):488–515. 10.17072/1995-4190-2021-53-488-515

[11] Turney J, Young TM, Chauhan DR, Beeharry R, Mahmud M. AI use for medical students: Impact on clinical skill acquisition and retention. A systematic review. Adv Med Educ Pract. 2026;17:583763. 10.2147/AMEP.S583763 42006163 PMC13084638

[12] Zheng C, Zhang H. Ethical issues in medical AI education: Challenges and prospects. Int J Ethics Educ. 2026. 10.1007/s40889-026-00241-y

[13] Garibaldi BT, Russell SW. Strategies to reinvigorate the bedside clinical encounter. N Engl J Med. 2025;393(21):2142–2150. 10.1056/NEJMra2500226 41223363

[14] Ke Y, Jin L, Ong JCL, et al. AI-induced never-skilling in medical education. Nat Med. 2026. 10.1038/s41591-026-04438-y 42174254

[15] Wosny M, Strasser LM, Hastings J. Experience of health care professionals using digital tools in the hospital: Qualitative systematic review. JMIR Hum Factors. 2023;10:e50357. 10.2196/50357 37847535 PMC10618886

[16] Fernandes Prabhu D, Gurupur V, Stone A, Trader E. Integrating artificial intelligence, electronic health records, and wearables for predictive, patient-centered decision support in healthcare. Healthcare. 2025;13(21):2753. 10.3390/healthcare13212753 41228120 PMC12607345

[17] Patocka C, Pandya A, Brennan E, et al. The impact of just-in-time simulation training for healthcare professionals on learning and performance outcomes: A systematic review. Simul Healthc. 2024;19(1S):S32–S40. 10.1097/SIH.0000000000000764 38240616

[18] Riel M. Education in the 21st century: just-in-time learning or learning communities. Paper presented at the Fourth Annual Conference of the Emirates Center for Strategic Studies and Research; 1998 May 24–26. Abu Dhabi: Emirates Center for Strategic Studies and Research, 2000; p. 137–160.

[19] Hennus MP, Ramani S, Van Dam M. Giving the patient a leading role in bedside teaching; a truly collaborative and inclusive effort. Med Teach. 2025;47(3):375–376. 10.1080/0142159X.2024.2398761 39225390

[20] Parmar R, Eldawud D, Sharma N, Issa R, Budzikowski A. Preserving bedside teaching: An AI-supported system for capturing spontaneous clinical teaching. Med Teach. 2026:1–4. 10.1080/0142159X.2025.2604243 41498442

[21] Ravichandran L, Saravanan S. Revitalizing the bedside teaching: The enduring value of clinical teaching at the patient’s side. Sri Ramachandra J Health Sci. 2025;5:1–2. 10.25259/SRJHS_9_2025

[22] Wu CJ, Raghavendra R, Gupta U, Acun B, Ardalani N, Maeng K, et al. Sustainable AI: environmental implications, challenges and opportunities. arXiv [Preprint]. 2022 [cited 2026 May 15]. Available from: https://arxiv.org/pdf/2111.00364.

[23] Franco D’Souza R, Mathew M, Mishra V, Surapaneni KM. Twelve tips for addressing ethical concerns in the implementation of artificial intelligence in medical education. Med Educ Online. 2024;29(1):2330250. 10.1080/10872981.2024.2330250 38566608 PMC10993743

[24] Hu L, Argus G, Pineda RC, MacAskill W, Martin P. The role of artificial intelligence in enhancing interprofessional education and collaborative practice: A mixed methods scoping review. J Interprof Care. 2026;40(2):390–399. 10.1080/13561820.2025.2576241 41347741

[25] Blumenthal D, Bennett K, McDonough J. Overcoming the digital divide in health care AI. N Engl J Med. 2026;394; 1876–1877. 10.1056/NEJMp2600816 42112869

[26] Ajalo E, Mukunya D, Nantale R, et al. Widespread use of ChatGPT and other Artificial Intelligence tools among medical students in Uganda: A cross-sectional study. PLoS One. 2025;20(1):e0313776. 10.1371/journal.pone.0313776 39787055 PMC11717177

[27] Nantale R, Kayemba F, Paungholi K, et al. Experiences of nursing and medical students with the use of Artificial Intelligence tools such as ChatGPT in Uganda. PLoS Glob Public Health. 2025;5(12):e0005715. 10.1371/journal.pgph.0005715 41468448 PMC12753054

[28] Mukunya D, Nantale R, Kayemba F, et al. Utilisation of ChatGPT and other Artificial Intelligence tools among medical faculty in Uganda: A cross-sectional study. MedEdPublish (2016). 2026;14:245. 10.12688/mep.20554.3 PMC11795020 39911314

[29] Titus S. Implementing extended reality (XR) and artificial intelligence (AI) in health professions education in southern Africa. Afr J Health Prof Educ. 2024;16(2):e1101. 10.7196/AJHPE.2024.v16i2.1101

[30] Grand Challenges. Transforming healthcare education: AI-powered access to clinical guidelines and standard operating procedures (SOPs) for health practitioners in Francophone Africa [homepage on the Internet]. Dakar, Senegal: Tech Care For All - Africa; 2024 [cited 2026 May 15]. Available from: https://gcgh.grandchallenges.org/grant/transforming-healthcare-education-ai-powered-access-clinical-guidelines-and-standard

[31] Smith SM, Tate M, Freeman K, Walsh A, Ballsun-Stanton B, Lane M. A university framework for the responsible use of generative AI in research. J High Educ Policy Manag. 2026;48(1):17–36. 10.1080/1360080X.2025.2509187

[32] Funa AA, Gabay RAE. Policy guidelines and recommendations on AI use in teaching and learning: A meta-synthesis study. Soc Sci Humanit Open. 2025;11:101221. 10.1016/j.ssaho.2024.101221

